# Selenium and Prostate Cancer: Analysis of Individual Participant Data From Fifteen Prospective Studies

**DOI:** 10.1093/jnci/djw153

**Published:** 2016-07-06

**Authors:** Naomi E. Allen, Ruth C. Travis, Paul N. Appleby, Demetrius Albanes, Matt J. Barnett, Amanda Black, H. Bas Bueno-de-Mesquita, Mélanie Deschasaux, Pilar Galan, Gary E. Goodman, Phyllis J. Goodman, Marc J. Gunter, Markku Heliövaara, Kathy J. Helzlsouer, Brian E. Henderson, Serge Hercberg, Paul Knekt, Laurence N. Kolonel, Christina Lasheras, Jakob Linseisen, E. Jeffrey Metter, Marian L. Neuhouser, Anja Olsen, Valeria Pala, Elizabeth A. Platz, Harri Rissanen, Mary E. Reid, Jeannette M. Schenk, Meir J. Stampfer, Pär Stattin, Catherine M. Tangen, Mathilde Touvier, Antonia Trichopoulou, Piet A. van den Brandt, Timothy J. Key, on behalf of the Endogenous Hormones, Nutritional Biomarkers and Prostate Cancer Collaborative Group

**Affiliations:** **Affiliations of authors:** Clinical Trial Service Unit and Epidemiological Studies Unit (NEA) and Cancer Epidemiology Unit (RCT, PNA, TJK), Nuffield Department of Population Health, University of Oxford, Oxford, UK; Division of Cancer Epidemiology and Genetics, US National Cancer Institute, Bethesda, MD (DA, AB); Division of Public Health Science (MJB, GEG, MLN), SWOG (formerly the Southwest Oncology Group) Statistical Center (PJG, CMT), and Cancer Prevention Program (JMS), Fred Hutchinson Cancer Research Center, Seattle, WA; Department of Epidemiology (JMS) and Department of Biostatistics (CMT), University of Washington, Seattle, WA; Department for Determinants of Chronic Diseases, National Institute for Public Health and the Environment (RIVM), Bilthoven, the Netherlands (HBBdM); Department of Gastroenterology and Hepatology, University Medical Centre, Utrecht, the Netherlands (HBBdM); Department of Epidemiology and Biostatistics, School of Public Health, Imperial College London, London, UK (HBBdM, MJG); Department of Social & Preventive Medicine, Faculty of Medicine, University of Malaya, Kuala Lumpur, Malaysia (HBBdM); Sorbonne Paris Cité Epidemiology and Biostatistics Research Center, Nutritional Epidemiology Research Team, Inserm U1153, Inra U1125, Cnam, University Paris 13, University Paris 5, University Paris 7, Bobigny, France (MD, PG, SH, MT); National Institute for Health and Welfare, Helsinki, Finland (MH, PK, HR); The Prevention and Research Center Mercy Medical Center, Baltimore, MD (KJH); Department of Preventive Medicine, Norris Comprehensive Cancer Center, Keck School of Medicine, University of Southern California, Los Angeles, CA (BEH); University of Hawaii Cancer Center, Honolulu, HI (LNK); Department of Functional Biology, Faculty of Medicine, University of Oviedo, Asturias, Spain (CL); Institute of Epidemiology II, Helmholtz-Zentrum München, Neuherberg, Germany (formerly of Division of Cancer Epidemiology, German Cancer Research Center, Heidelberg, Germany) (JL); Intramural Research Program, National Institute on Aging, Department of Neurology, University of Tennessee Health Science Center, Memphis, TN (EJM); Danish Cancer Society Research Center, Copenhagen, Denmark (AO); Epidemiology and Prevention Unit, Fondazione IRCCS Istituto Nazionale dei Tumori, 20133 Milan, Italy (VP); Department of Epidemiology, Johns Hopkins Bloomberg School of Public Health, Baltimore, MD (EAP); Roswell Park Cancer Institute, New York, NY (MER); Department of Epidemiology, Harvard School of Public Health, Boston, MA (MJS); Channing Division of Network Medicine, Brigham and Women’s Hospital, Harvard Medical School, Boston, MA (MJS); Department of Surgery and Perioperative Sciences, Urology and Andrology, Umeå University, Umeå, Sweden (PS); Hellenic Health Foundation, Athens, Greece (AT); Department of Epidemiology, GROW School for Oncology and Developmental Biology, Maastricht University, the Netherlands (PAvdB)

## Abstract

**Background:** Some observational studies suggest that a higher selenium status is associated with a lower risk of prostate cancer but have been generally too small to provide precise estimates of associations, particularly by disease stage and grade.

**Methods:** Collaborating investigators from 15 prospective studies provided individual-participant records (from predominantly men of white European ancestry) on blood or toenail selenium concentrations and prostate cancer risk. Odds ratios of prostate cancer by selenium concentration were estimated using multivariable-adjusted conditional logistic regression. All statistical tests were two-sided.

**Results:** Blood selenium was not associated with the risk of total prostate cancer (multivariable-adjusted odds ratio [OR] per 80 percentile increase = 1.01, 95% confidence interval [CI] = 0.83 to 1.23, based on 4527 case patients and 6021 control subjects). However, there was heterogeneity by disease aggressiveness (ie, advanced stage and/or prostate cancer death, *P*_heterogeneity_ = .01), with high blood selenium associated with a lower risk of aggressive disease (OR = 0.43, 95% CI = 0.21 to 0.87) but not with nonaggressive disease. Nail selenium was inversely associated with total prostate cancer (OR = 0.29, 95% CI = 0.22 to 0.40, *P*_trend_ < .001, based on 1970 case patients and 2086 control subjects), including both nonaggressive (OR = 0.33, 95% CI = 0.22 to 0.50) and aggressive disease (OR = 0.18, 95% CI = 0.11 to 0.31, *P*_heterogeneity_ = .08).

**Conclusions:** Nail, but not blood, selenium concentration is inversely associated with risk of total prostate cancer, possibly because nails are a more reliable marker of long-term selenium exposure. Both blood and nail selenium concentrations are associated with a reduced risk of aggressive disease, which warrants further investigation.

There has been great interest in the possible role of selenium in cancer prevention, largely because of its antioxidant properties, although it also has other potentially anticarcinogenic effects (1). The efficacy of selenium as a cancer prevention agent has been tested in several randomized controlled trials although results have been inconsistent, perhaps because of the relatively small number of cases (particularly of aggressive disease) identified during follow-up and different levels of baseline selenium in the populations under study (2–6). Results from observational studies are also conflicting (7–20); some have reported high blood or nail selenium to be associated with a lower risk of total prostate cancer (8,9) or aggressive disease (10–12) while others have found no statistically significant association (13–20).

The Endogenous Hormones, Nutritional Biomarkers and Prostate Cancer Collaborative Group (EHNBPCCG) was established to conduct collaborative analyses of individual participant data from prospective studies on the associations of circulating concentrations of hormones and nutritional biomarkers with the subsequent risk of prostate cancer (21–26). The objective of the present study is to determine the association between blood and nail selenium concentration and risk of prostate cancer in 15 prospective studies and to evaluate this association by stage and grade of disease and other characteristics.

## Methods

### Identification of Studies and Collection of Data

A detailed description of the EHNBPCCG is reported elsewhere (21). In 2010, collaborators were invited to contribute data on prediagnostic measures of nutritional biomarkers and prostate cancer risk. Studies were identified from review articles, literature searches using PubMed, Web of Science, Cochrane Library, and CancerLit (up to January 2013), and from discussions with colleagues in order to identify unpublished data.

Seventeen eligible studies were identified, of which 15 are included in the current analysis, comprising 6497 men with prostate cancer (case patients) and 8107 men without prostate cancer (control subjects) (2, 4, 8, 10–19), including unpublished data from the Prostate Cancer Prevention Trial (PCPT), provided with kind permission by Phyllis Goodman, Catherine Tangen, and Jeannette Schenk from the Fred Hutchinson Cancer Research Center (Seattle, WA); and from the SUpplémentation en VItamines et Minéraux Anti-oXydants (SU.VI.MAX) Trial by Pilar Galan, Mélanie Deschasaux, and Mathilde Touvier, from Inserm (University of Paris, Paris, France), of which study details are reported elsewhere (27,28). Data were not available for the Uppsala Longitudinal Study of Adult Men (ULSAM) (20) or the Honolulu Heart Program (9).

Information on recruitment, informed consent, ethical approval, and inclusion criteria for all studies is available in the original publications. Eight studies used a nested case-control study design in populations with no organized screening program but with varying levels of population-wide prostate-specific antigen (PSA) testing (8,11,14–19); seven studies were observational investigations based within randomized controlled trials (2,4,10,13,16,27,28) (Supplementary Table 1, available online).

Individual participant data were requested for selenium concentrations measured in blood or nails (see Supplementary Table 2, available online, for details of the assay methods), date and age at sample collection, fasting status, marital status, ethnicity, educational attainment, family history of prostate cancer, height, weight, waist and hip circumference, smoking status, and alcohol intake. Most studies included men of white European ancestry; those studies that did include men of different ethnic backgrounds included this as a matching criterion (4,14,18).

Each study provided data on prostate cancer stage and grade, if available. Tumor stage was categorized as localized if it was TNM staging tumor (T)–node (N)-metastasis (M) categories of T2 or lower with no reported lymph node involvement or metastases, stage II or lower or equivalent; advanced if it was T3 or T4 and/or N1+ and/or M1, stage III or IV or equivalent; or stage unknown. Aggressive disease was defined as T4 and/or N1+ and/or M1, or stage IV disease and/or death from prostate cancer; nonaggressive disease was defined as TNM stage lower than T4 with no reported lymph node involvement or metastases or the equivalent, or unknown. Histological grade was categorized as low-intermediate grade (Gleason sum < 8, or coded as well, moderately, or poorly differentiated), high-grade (Gleason sum 8+, or coded as undifferentiated), or unknown. Our definition of high-grade disease used in this and other analyses (23–26) is more stringent than our previous collaborative analyses (which used Gleason score of 7 or above) (21,22); while this will lead to identification of relatively small numbers of high-grade tumours, it reduces the risk of misclassification of high-grade disease.

### Statistical Analysis

Blood and nail selenium concentrations were logarithmically transformed for all statistical analyses to approximate a normal distribution. All analyses were run separately for blood and nail selenium. Analysis of covariance was used to investigate geometric mean differences in selenium concentration among the controls by baseline characteristics, with adjustment for study and age at blood collection. Tests for trend across the categories were obtained by scoring the categories 1, 2, 3, etc., as required.

Men were categorized into fifths of selenium concentration with quintile cutpoints defined by the overall distribution among control participants in all studies combined. This approach maximizes the ability to examine associations across the full selenium distribution across all studies and assumes that the differences in absolute values between studies are because of true population differences rather than assay differences between the studies (29). Analyses were also performed with cutpoints defined by the study-specific quintiles of selenium concentration.

To provide a summary measure of risk, a linear trend was calculated by replacing the categorical variable representing the fifths of selenium with a continuous variable that was scored as 0, 0.25, 0.5, 0.75, and 1. As the midpoints of the lowest and highest fifths are the 10th and 90th percentiles of the study-specific selenium concentration, a unit change in this continuous trend variable (which represents an 80th percentile increase) is comparable with the relative risk comparing the highest with the lowest fifth.

Conditional logistic regression was used to calculate odds ratios (ORs) and their corresponding 95% confidence intervals (CIs), conditioned on the matching variables within each study and with additional adjustment for age at blood collection (exact), body mass index (BMI = <25, 25–27.4, 27.5–29.9, ≥30 kg/m^2^, not known), height (≤170, 171–175, 176–180, >180 cm, not known), marital status (married/cohabiting, not married/cohabiting, not known), educational status (did not graduate from high school/secondary school/college, high school/secondary school/college graduates, university graduates, not known), and cigarette smoking (never, past, current, not known). These factors were found to be associated with prostate cancer risk in this dataset and hence were included in the final models.

Heterogeneity in linear trends between studies was tested by comparing the χ^2^ values for models with and without a (study) x (linear trend) interaction term. Tests for heterogeneity of trends for the case-defined factors were obtained by fitting separate models for each subgroup and assuming independence of the odds ratios using a method analogous to a meta-analysis. Tests for heterogeneity for the non-case-defined factors were assessed with a χ^2^-test of interaction between subgroup and the continuous trend test variable. Further analyses of the association of selenium with disease stage, aggressiveness, and grade were conducted by calculating odds ratios in fifths of the distribution.

All *P* values reported are two-tailed, and a *P* value of less than .05 was considered statistically significant. All statistical analyses were carried out using Stata (StataCorp LP, 2011, Stata Statistical Software: Release 13, College Station, TX).

## Results

Details of the 15 participating studies are shown in Table 1. Data were available for 4527 case patients and 6021 control subjects for blood selenium and for 1970 cases and 2086 controls for nail selenium. In European populations, median values in controls were below 1130 nmol/L and 0.55 ppm for blood and nail samples, respectively; studies in the United States had median values higher than 1370 nmol/L and higher than 0.78 ppm, respectively (Table 1). The duration of follow-up varied substantially; in some studies (Baltimore Longitudinal Study of Aging [BLSA], beta-Carotene and Retinol Efficacy Trial [CARET], Multi-Ethnic Cohort [MEC], Prostate, Lung, Colorectal and Ovarian Cancer Screening trial [PLCO], and Selenium and Vitamin E Cancer Prevention Trial [SELECT]), more than 70% of men were diagnosed less than five years following recruitment; in others (European Prospective Investigation into Cancer and Nutrition-Heidelberg [EPIC-Heidelberg], Netherlands Cohort Study [NLCS], PCPT, Physicians’ Health Study [PHS] and SU.VI.MAX), more than 70% of case patients were diagnosed five or more years after recruitment (Table 2). Age at diagnosis varied less widely, although the SU.VI.MAX trial included a higher proportion of men diagnosed at an early age (34% of case patients were diagnosed at younger than age 60 years) compared with the other studies. The time period of diagnosis varied substantially between studies owing to the different time periods of recruitment and the duration of follow-up. The proportion of case patients with advanced disease varied from 1.6% (PCPT) to 72% (NLCS). The proportion of case patients with high-grade disease varied from 0.2% (MEC) to 16% (HPFS) (Table 2; see Supplementary Table 3, available online, for details of the number of cases by stage, grade, and other tumor characteristics by study).

**Table 1.** Details of the studies and participants included*

Study	No.		Mean (SD) age at sample collection, y		Median (IQR) selenium concentration (nmol/L for blood; ppm for nails)	
	Case patients	Control subjects	Case patients	Control subjects	Case patients	Control subjects
Blood selenium						
BLSA (8)	55	55	69.1 (8.6)	68.8 (7.7)	1431 (1317–1671)	1532 (1418–1684)
CARET (13)	235	456	61.5 (6.1)	61.3 (6.2)	1462 (1280–1599)	1425 (1282–1595)
EPIC (17)	959	1,059	59.9 (5.8)	59.6 (5.8)	898 (791–1022)	910 (809–1027)
EPIC-Heidel (19)	148	291	57.4 (5.1)	57.8 (5.1)	1100 (1010–1230)	1100 (994–1210)
FMC (15)	51	92	65.5 (7.7)	65.4 (7.4)	709 (582–912)	734 (595–893)
MEC (18)	461	920	69.0 (7.0)	68.8 (7.1)	1708 (1582–1865)	1718 (1594–1871)
NPC (2)	41	123	66.9 (5.0)	66.8 (4.9)	1433 (1256–1570)	1433 (1241–1605)
PCPT (unpublished)	960	960	63.4 (5.5)	63.3 (5.6)	1687 (1544–1845)	1660 (1529–1830)
PHS (10)	794	794	59.0 (8.2)	58.8 (8.1)	1374 (1221–1532)	1371 (1223–1545)
PLCO (16)	723	879	65.1 (4.8)	64.8 (4.7)	1775 (1589–1946)	1797 (1605–2000)
SU.VI.MAX (unpublished)	100	392	55.1 (4.6)	55.0 (4.6)	1115 (1026–1252)	1127 (995–1254)
Nail selenium						
CLUE II (14)	117	233	65.9 (7.5)	65.9 (7.5)	0.77 (0.68–0.86)	0.79 (0.70–0.87)
HPFS (11)	181	181	63.2 (6.4)	63.1 (6.4)	0.79 (0.70–0.91)	0.80 (0.73–0.94)
NLCS (12, 49)	1268	1268	62.7 (4.1)	62.7 (4.1)	0.51 (0.46–0.57)	0.54 (0.48–0.60)
SELECT (4)	404	404	63.3 (6.0)	62.7 (4.1)	0.87 (0.79–0.97)	0.88 (0.77–0.99)

* The numbers of case patients and control subjects are the number for whom selenium measurements were available. BLSA = Baltimore Longitudinal Study of Aging; CARET = the beta-Carotene and Retinol Efficacy Trial; CLUE II = Campaign against Cancer and Stroke (“Give us a Clue to Cancer”) Study; EPIC = European Prospective Investigation into Cancer and Nutrition; EPIC-Heidel = EPIC-Heidelberg; FMC = Finnish Mobile Clinic Health Examination Survey; HPFS = Health Professionals Follow-up Study; MEC = Multiethnic Cohort; NLCS = Netherlands Cohort Study; NPC = Nutritional Prevention of Cancer Trial; PCPT = Prostate Cancer Prevention Trial; PHS = Physicians’ Health Study; PLCO = Prostate, Lung, Colorectal and Ovarian Cancer Screening Trial; SELECT = Selenium and Vitamin E Cancer Prevention Trial; SU.VI.MAX = SUpplémentation en VItamines et Minéraux Anti-oXydants Trial.

**Table 2.** Proportions of men with prostate cancer by selected characteristics in each study*

Study	No. of case patients	Years from sample collection to diagnosis, %		Age at diagnosis, %			Year of diagnosis, %		Stage of disease†, %			Aggressive disease†, %			Grade†, %		
		<5	5+	<60 y	60-69 y	70+ y	<1995	1995+	Loc	Adv	N/k	No	Yes	N/k	Low-interm	High	N/k
Blood selenium																	
BLSA (8)	55	70.9	29.1	3.6	27.3	69.1	74.5	25.5	–	–	100	0	12.7	87.3	78.2	7.3	14.5
CARET (13)	235	70.2	29.8	14.0	56.2	29.8	21.3	78.7	50.2	21.3	28.5	62.1	11.9	26.0	74.5	11.9	13.6
EPIC (17)	959	63.1	36.9	19.5	65.0	15.5	0.4	99.6	51.8	21.2	27.0	53.7	24.8	21.5	66.6	10.6	22.7
EPIC-Heidel (19)	148	23.0	77.0	16.2	71.6	12.2	0	100	76.4	23.0	0.7	92.6	6.8	0.6	89.2	9.5	1.4
FMC (15)	51	45.1	54.9	9.8	41.2	49.0	100	0	–	–	100	–	–	100	–	–	100
MEC (18)	461	93.7	6.3	7.2	33.4	59.4	0	100	–	–	100	0	5.6	94.4	95.2	0.2	4.6
NPC (2)	41	56.1	43.9	0	43.9	56.1	92.7	7.3	75.6	24.4	0	82.9	17.1	0	82.9	14.6	2.4
PCPT (unpublished)	960	21.3	78.7	1.6	50.5	47.9	0.1	99.9	95.7	1.6	2.7	96.5	0.8	2.7	93.0	4.7	2.3
PHS (10)	794	15.1	84.9	12.0	44.6	43.5	76.8	23.2	79.8	15.0	5.2	74.6	20.9	4.5	86.4	10.3	3.3
PLCO (16)	723	88.9	11.1	5.5	55.9	38.6	0	100	87.1	12.9	0	93.1	6.9	0	93.5	5.9	0.6
SU.VI.MAX (unpublished)	100	28.0	72.0	34.0	66.0	0	0	100	–	–	100	–	–	100	84.0	10.0	6.0
Nail selenium																	
CLUE II (14)	117	67.5	32.5	8.5	36.8	54.7	73.5	26.5	53.8	26.5	19.7	65.0	18.8	16.2	85.5	6.8	7.7
HPFS (11)	181	63.0	37.0	11.0	46.4	42.5	99.4	0.6	51.9	43.6	4.4	54.1	43.7	2.2	60.8	16.0	23.2
NLCS (12, 49)	1268	29.1	70.9	1.0	38.0	61.0	53.2	46.8	21.3	72.2	6.5	47.0	47.5	5.5	91.0	0.9	8.1
SELECT (4)	404	78.7	21.3	12.6	55.7	32.7	0	100	98.0	0	2.0	97.3	0.7	2.0	82.4	4.7	12.9

* The number of case patients in each of these categories by study is shown in Supplementary Table 3 (available online). BLSA = Baltimore Longitudinal Study of Aging; CARET = the beta-Carotene and Retinol Efficacy Trial; CLUE II = Campaign against Cancer and Stroke ("Give us a Clue to Cancer") Study; EPIC = European Prospective Investigation into Cancer and Nutrition; EPIC-Heidel = EPIC-Heidelberg; FMC = Finnish Mobile Clinic Health Examination Survey; HPFS = Health Professionals Follow-up Study; MEC = Multiethnic Cohort; NLCS = Netherlands Cohort Study; NPC = Nutritional Prevention of Cancer Trial; PCPT = Prostate Cancer Prevention Trial; PHS = Physicians’ Health Study; PLCO = Prostate, Lung, Colorectal and Ovarian Cancer Screening Trial; SELECT = Selenium and Vitamin E Cancer Prevention Trial; SU.VI.MAX = SUpplémentation en VItamines et Minéraux Anti-oXydants Trial.

^†^Stage of disease was defined as: localized if TNM was T2 or lower with no reported lymph node involvement or metastases, stage II or lower, or equivalent (ie, a tumor that does not extend beyond the prostate capsule); advanced if TNM stage was T3 or T4 and/or N1+ and/or M1, stage III or IV, equivalent (ie, a tumor extending beyond the prostate capsule and/or lymph node involvement and/or distant metastases), or unknown. Overall, 5315 (82%) of case patients had data on stage. Aggressive disease was defined as T4 and/or N1+ and/or M1+, or stage IV disease and/or death from prostate cancer. Overall, 5432 (84%) of case patients had data on disease aggressiveness.

^‡^Histological grade was categorized as low-intermediate grade (Gleason sum <8 or cases coded as well, moderately, or poorly differentiated), high-grade (Gleason sum 8+ or cases coded as undifferentiated), or unknown. Overall, 5900 (91%) of case patients had information on grade.

The cross-sectional associations between baseline characteristics and blood and nail selenium concentrations are shown in Supplementary Figure 1 (available online). Blood selenium concentrations were slightly lower in men who were older, who had a higher BMI, and who were current smokers and were slightly higher in men who had fasted for longer at the time of blood collection. Nail selenium concentrations were lower in current smokers and slightly higher in men who were married and highly educated.

**Figure 1.**
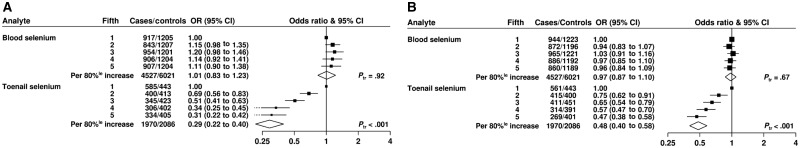
Odds ratios (95% confidence intervals [CIs]) of prostate cancer associated with fifths of blood and nail selenium concentration, adjusted for age at blood collection, body mass index, height, marital status, education, and smoking. The *P*_trend_ was calculated by replacing the fifths of selenium with a continuous variable that was scored as 0, 0.25, 0.5, 0.75, and 1 in the conditional logistic regression model. Median concentrations in each fifth (using overall cutpoints) are: 874, 1184, 1467, 1677, and 1939 nmol/L for blood selenium and 0.46, 0.54, 0.63, 0.77, and 0.96 ppm for nail selenium. All statistical tests were two-sided. Results in the figures are presented as **squares** and **lines**, representing the odds ratios and corresponding 95% confidence intervals, respectively. The **position of the square** indicates the value of the odds ratio while the **size of the square** is inversely proportional to the variance of the logarithm of the odds ratio and indicates the amount of statistical information available for that particular estimate. The **open diamonds** (the lateral points of which are the 95% CIs) represent the overall odds ratio for an 80th percentile increase in selenium concentration. 80%le = 80 percentile; CI = confidence interval; OR = odds ratio; P_tr_ = *P*_trend_.

The associations of blood and nail selenium concentrations with prostate cancer risk are shown in Figure 1A. After adjustment for BMI, height, age, marital status, education, and smoking status, the odds ratio per 80th percentile increase of blood selenium concentration was 1.01 (95% CI = 0.83 to 1.23, *P*_trend_ = .92), based on 4527 case patients and 6021 control subjects; for nail selenium, the corresponding odds ratio was 0.29 (95% CI = 0.22 to 0.40, *P*_trend_ < .001), based on 1970 case patients and 2086 control subjects. Adjustment for the potential confounders listed above made little difference to the risk estimates (unadjusted OR per 80 percentile increase was 1.02, 95% CI = 0.85 to 1.24, *P*_trend_ = 0.81 for blood selenium; OR = 0.36, 95% CI = 0.27 to 0.48, *P*_trend_ < .001 for nail selenium). Associations were qualitatively similar when the analysis was performed using study-specific cutpoints although the risk estimate was attenuated for nail measures, reflecting the narrower ranges of selenium concentration when using these cutpoints (Figure 1B).

In order to examine the association at the extremes of the distribution, selenium concentration was categorized into deciles and analyzed using overall cutpoints for all studies combined. There remained no association with blood selenium (multivariable-adjusted OR for the highest [midpoint = 2092 nmol/L] vs lowest tenth [midpoint = 792 nmol/L] = 0.94, 95% CI = 0.71 to 1.23, *P*_trend_ = .69) and a strong inverse association with nail selenium (OR for the highest [midpoint = 1.08 ppm] vs lowest tenth (midpoint = 0.17 ppm) = 0.25, 95% CI = 0.17 to 0.37, *P*_trend_ < .001).

**Figure 2.**
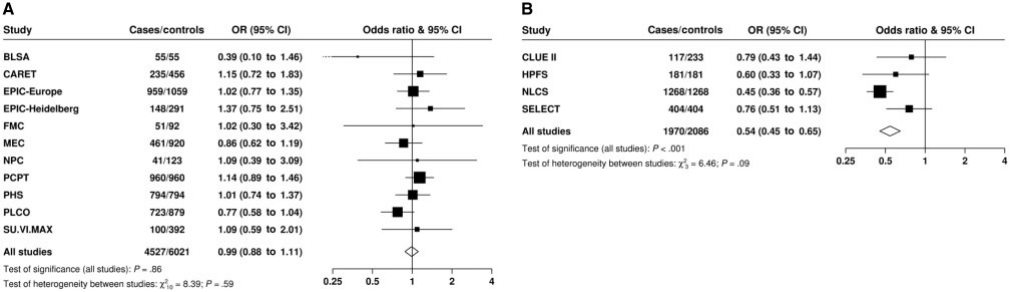
Study-specific odds ratios (95% confidence intervals [CIs]) of prostate cancer per 80th percentile increase in **(A)** blood and **(B)** nail selenium concentration. The odds ratios are calculated by conditioning on the matching variables within each study (but not further adjusted). Heterogeneity in linear trends between studies was tested by comparing the χ2 values for models with and without a (study) x (linear trend) interaction term. All statistical tests were two-sided. Results in the figures are presented as **squares** and **lines**, representing the odds ratios and corresponding 95% confidence intervals, respectively. The **position of the square** indicates the value of the odds ratio while the **size of the square** is inversely proportional to the variance of the logarithm of the odds ratio and indicates the amount of statistical information available for that particular estimate. The **open diamonds** (the lateral points of which are the 95% CIs) represent the overall odds ratio for an 80th percentile increase in selenium concentration. BLSA = Baltimore Longitudinal Study of Aging; CARET = the beta-Carotene and Retinol Efficacy Trial; CI = confidence interval; CLUE II = Campaign against Cancer and Stroke ("Give us a Clue to Cancer") Study; EPIC = European Prospective Investigation into Cancer and Nutrition; EPIC-Heidel = EPIC-Heidelberg; FMC = Finnish Mobile Clinic Health Examination Survey; HPFS = Health Professionals Follow-up Study; MEC = Multiethnic Cohort; NLCS = Netherlands Cohort Study; NPC = Nutritional Prevention of Cancer Trial; PCPT = Prostate Cancer Prevention Trial; PHS = Physicians’ Health Study; PLCO = Prostate, Lung, Colorectal and Ovarian Cancer Screening Trial; SELECT = Selenium and Vitamin E Cancer Prevention Trial; SU.VI.MAX = SUpplémentation en VItamines et Minéraux Anti-oXydants Trial.

There was no evidence of heterogeneity between the contributing studies in the linear association of blood selenium concentration with risk (*P*_heterogeneity_ = .59) (Figure 2A). There was also no evidence of heterogeneity in the association of nail selenium concentration with risk between the studies (*P*_heterogeneity_ = .09) (Figure 2B), and the inverse association with prostate cancer persisted after exclusion of the largest dataset (NLCS) although it was attenuated (OR per 80th percentile increase = 0.69, 95% CI = 0.50 to 0.94, *P*_trend_ = .02).

**Figure 3.**
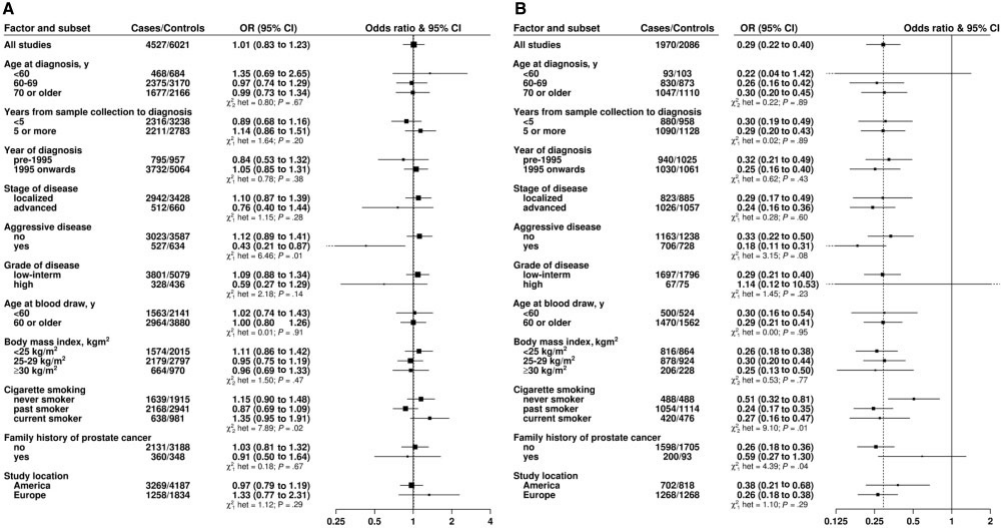
Odds ratios (95% confidence intervals [CIs]) of prostate cancer associated with an 80th percentile increase across all studies combined in **(A)** blood and **(B)** nail selenium concentration in selected subgroups, adjusted for age at blood collection, body mass index, height, marital status, education, and smoking. Tests for heterogeneity for the case-defined factors were obtained by fitting separate models for each subgroup and assuming independence of the odds ratios using a method analogous to a meta-analysis. Tests for heterogeneity for the non-case-defined factors were assessed with a χ2-test of interaction between subgroup and the continuous trend test variable. All statistical tests were two-sided. Results in the figures are presented as **squares** and **lines**, representing the odds ratios and corresponding 95% confidence intervals, respectively. The **position of the square** indicates the value of the odds ratio while the **size of the square** is inversely proportional to the variance of the logarithm of the odds ratio and indicates the amount of statistical information available for that particular estimate. The **open diamonds** (the lateral points of which are the 95% CIs) represent the overall odds ratio for an 80th percentile increase in selenium concentration. CI = confidence interval; het = heterogeneity; OR = odds ratio.

There was evidence of heterogeneity in the linear association of blood selenium concentration with prostate cancer risk according to disease aggressiveness (*P*_heterogeneity_ = .01), with a higher blood selenium concentration associated with a lower risk of aggressive disease (OR per 80th percentile increase = 0.43, 95% CI = 0.21 to 0.87), with no association with nonaggressive disease (OR = 1.12, 95% CI = 0.89 to 1.41) (Figure 3A). The differences in associations of blood selenium with risk by stage and grade of disease were not statistically significant although a reduced risk with high blood selenium was also seen for men diagnosed with advanced-stage or high-grade disease. Analyses of the relationship between overall fifths of blood selenium concentration and risk of prostate cancer by disease stage, aggressiveness, and grade are shown in Supplementary Figure 2 (available online). These findings support the linear trend estimates although the numbers are small in some categories. There were no statistically significant differences in the association between nail selenium concentration and prostate cancer risk by stage or grade of disease, although nail selenium was associated with a particularly low risk for aggressive disease (OR per 80th percentile increase = 0.18, 95% CI = 0.11 to 0.31) compared with nonaggressive disease (OR = 0.33, 95% CI = 0.22 to 0.50, *P*_heterogeneity_ = .08) (Figure 3B).

Of the eight other factors examined, there was also evidence of heterogeneity in the linear association of both blood and nail selenium concentration with prostate cancer risk by smoking status (*P*_heterogeneity_ = .02 and .01, respectively) and for nail selenium by family history (*P*_heterogeneity_ = .04) (Figure 3).

## Discussion

This international collaboration has brought together and re-analyzed almost all of the available data on the association of blood and nail selenium concentrations and prostate cancer incidence. The majority of the data were from studies with blood measures, which showed no association with risk of total prostate cancer (based on 4527 case patients and 6021 control subjects) but an approximate halving in risk for aggressive disease in men with high blood concentrations (based on 527 case patients and 634 control subjects). Combining (using a weighted average) our collaborative risk estimate with those from the two blood-based studies that were unable to contribute data (9,20) produced similar findings (OR for high vs low category of selenium concentration = 0.92, 95% CI = 0.82 to 1.04).

Four studies provided data on nail measures, which showed a 70% reduction in risk for total prostate cancer (based on 1970 case patients and 2086 control subjects), with no statistically significant difference by disease subgroups, although the association was somewhat stronger for aggressive compared with nonaggressive disease. The majority of the data are from a single study (NLCS), which also had substantially lower selenium concentrations than the other studies and a high proportion of advanced and aggressive disease, owing to an enriched sampling of such cases.

Our overall finding of a null association of total prostate cancer risk with blood selenium levels is consistent with findings from the SELECT trial, which randomly assigned men to selenium supplementation (200 µg/day L-selenomethionine) with or without vitamin E (400 IU/d of *all rac-*α*-*tocopheryl acetate) for 5.5 years, based on 848 cases (3). Two smaller randomized trials of selenium supplementation among men at a high risk of developing prostate cancer (the SWOG S9917 and the Negative Biopsy Trial) also showed no association with prostate cancer risk after three to five years follow-up although these findings were based on small numbers of cases (97 and 74, respectively) (5,6). It has been argued that the lack of a protective effect may have been because of the relatively high baseline serum selenium concentration in these men (with a median of 1715 nmol/L in the SELECT trial). This is because the NPC trial, designed to assess the efficacy of selenium supplementation for the prevention of nonmelanoma skin cancer, found that selenium supplementation (as 200 µg/d selenium in the form of selenized yeast) for an average of 4.5 years was associated with a statistically significant 52% reduction in prostate cancer risk 13 years later (based on 63 cases), which was particularly evident among men with a low baseline serum concentration (ie, <1290 nmol/L at entry to the trial) (2).

The apparent difference in risk observed for total prostate cancer between the blood and nail measures may partly be because of the differences in case-mix across the studies. Compared with studies with blood measures, those with nail measures contained a higher proportion of advanced-stage (55% vs 14%) and aggressive disease (38% vs 15%) and more men whose samples were taken before 1995 (48% vs 18%). Widespread use of PSA testing was introduced in many countries from the mid-1990s onwards (although use has varied between populations) while PSA screening was used systematically in two of the collaborating studies (PCPT and PLCO), contributing to an increase in the detection of small, asymptomatic tumors in recent years (30).

Because a substantial proportion of PSA-detected cancers remain biologically indolent for many years (31), the identification of factors that are associated with the development of clinically aggressive cancers is important. Hence, our finding that both blood and nail concentrations were associated with a lower risk of aggressive prostate cancer is of potential etiological relevance. However, there are some data to suggest that, among men diagnosed with nonmetastatic disease, high doses of selenium supplementation (140 µg/day or more) might be associated with an increased risk of prostate cancer death (32). Moreover, the SELECT trial also found that selenium supplementation was associated with an increased risk of high-grade (Gleason score ≥7) prostate cancer among men with high baseline selenium levels but not among men with lower baseline nail levels (4). However, the results of trials of the effect of selenium supplementation on PSA velocity (used as a marker of disease progression) have been inconsistent (6,33). Overall, owing to the low numbers of aggressive cancers diagnosed in many populations (in the current analyses, SELECT and NPC contributed 3 and 7 cases of aggressive disease, respectively), trials of selenium supplementation for the primary prevention of prostate cancer would have to be very large to assess differences by tumor stage and grade and are unlikely to be funded. Rather, further prospective studies in populations with a relatively high incidence of aggressive disease are warranted to investigate the association of long-term selenium concentration with disease incidence and progression.

Although it is possible that the inverse association found with blood and nail selenium concentration and risk of aggressive prostate cancer might be because of confounding, the analyses were adjusted for a range of lifestyle factors in addition to the matching factors used in the individual studies, none of which materially influenced the risk estimates. There was also no statistically significant heterogeneity in the association of blood or nail selenium concentration with risk of prostate cancer according to the number of years between blood collection and diagnosis, which does not suggest that reverse causation has affected the results, although this cannot be excluded. Finally, while there was some evidence of statistical heterogeneity in the association of selenium with risk by smoking status, the pattern of the association differed between blood and nail measures and the statistically significant heterogeneity may have been because of chance because of the large number of statistical tests performed.

Relatively little is known about the performance of blood and nail specimens as measures of long-term selenium status. Studies have shown that both blood and nail measures are correlated with selenium intake (r = 0.6 to 0.7) (34, 35) and with each other (r = 0.6) (36). Nail clippings provide a measure of exposure over several weeks up to six to 12 months before sample collection (37), with good repeatability over several years (r = 0.5 to 0.7) (38–40). Blood levels represent shorter-term selenium exposure (1–2 weeks) (37), although we have not identified data on the reproducibility of circulating concentrations to determine its stability over the long term. Thus, the differences in the findings for total prostate cancer between blood and nail measures may suggest that longer-term measures of selenium exposure are more relevant. Nonetheless, it remains difficult to determine the extent to which circulating or nail concentrations accurately reflect biological activity within prostatic tissue (41), especially at higher levels of selenium intake, when glutathione peroxidase activity is saturated (42). Genetic studies may help to clarify the role of individual selenoproteins in prostate carcinogenesis (19,43–48) although more work is needed to elucidate the relationship of variants in genes that help to regulate selenium status with levels of functional biomarkers of selenium and the influence of such variants as modifiers on the relationship between selenium and prostate cancer risk.

This collaborative re-analysis of the association of selenium status and prostate cancer risk based on the totality of the worldwide data shows that relatively high blood and nail selenium concentrations are associated with a reduced risk of aggressive prostate cancer. Further investigation in large, representative populations that have selenium measurements from both blood and nail samples and that include information on screening history, as well as stage and grade of tumors, is needed to examine these possible associations in more detail.

## Funding

This work was supported by Cancer Research UK (grant numbers C570/A11691, C8221/A19170). We thank the men who participated in the collaborating studies, the research staff, the collaborating laboratories, and the funding agencies in each of the studies. BLSA was supported by the National Institute on Aging Intramural Research Program, Prostate Spore Grant (CA58236). CARET was supported by the US Department of Health and Human Services and the National Institutes of Health (U01 CA63673), Clinical Nutrition Research Unit (P30 DK35816). Campaign against Cancer and Stroke was supported by the US National Cancer Institute, the National Institutes of Health (CA94028), and the Department of Defense (DAMD1–94-J-4265). European Investigation into Cancer and Nutrition was supported by Cancer Research UK; Europe Against Cancer Programme of the European Commission; German Cancer Aid; German Cancer Research Center; German Federal Ministry of Education and Research; Danish Cancer Society; Health Research Fund of the Spanish Ministry of Health; Centros de Investigacion Biomedica en Red Epidemiología y Salud Publica, Barcelona, Spain; the participating regional governments and institutions of Spain; Medical Research Council, UK; the Stroke Association (UK); British Heart Foundation; Department of Health (UK); Food Standards Agency (UK); Greek Ministry of Education; Greek Ministry of Health and Social Solidarity; Hellenic Health Foundation; Italian Association for Research on Cancer; Italian National Research Council; Dutch Ministry of Public Health, Welfare and Sports; Dutch Ministry of Health; Dutch Prevention Funds; LK Research Funds; Dutch Zorg Onderzoek Nederland; World Cancer Research Fund; Swedish Cancer Society; Swedish Scientific Council; and Regional Government of Skane, Sweden. EPIC-Heidelberg was supported by the German Federal Ministry of Education and Research (FK 0313846A), Deutsche Krebshilfe (10-1793 Scholl). Finnish Mobile Clinic Health Examination was supported by the Cancer Society of Finland. Health Professionals Follow-up Study was supported by the US National Cancer Institute, the National Institutes of Health (CA55075), and the National Heart, Lung, and Blood Institute (HL35464). Multiethnic Cohort was supported by the US National Cancer Institute and the National Institutes of Health (P01 CA 33619, R37 CA 54281). Nutritional Prevention of Cancer Trial was supported by the US National Cancer Institute and the National Institutes of Health (RO1 CA49764). Netherlands Cohort Study was supported by the Dutch Cancer Society (UM 2009–4556). Prostate Cancer Prevention Trial and Selenium and Vitamin E Cancer Prevention Trial (SouthWest Oncology Group) were supported by the US National Cancer Institute and the National Institutes of Health (CA37429). Physicians’ Health Study was supported by the US National Cancer Institute and the National Institutes of Health (CA42182, CA58684, CA57374). Prostate, Lung, Colorectal and Ovarian Cancer Screening Trial was supported by the US National Cancer Institute, the National Institutes of Health (PLCO Cancer Screening Trial), and the Intramural Research Program. SUpplementation en Vitamines et Mineraux Anti-oXydants Trial was supported by the French Ministry of Health/Direction Générale de la Santé.

## Notes

The authors are solely responsible for the study design; the collection, analysis, and interpretation of the data; the writing of the article; and the decision to submit the article for publication. All authors contributed to these analyses and read and approved the manuscript. The sponsors had no role in the study design, data collection, data analysis, data interpretation, writing of the report, or the decision to publish. The authors have no conflicts of interest to declare.

## Supplementary Material

Supplementary Data
