# Supplementary material for: Selenium and Prostate Cancer: Analysis of Individual Participant Data From Fifteen Prospective Studies
Source: J Natl Cancer Inst. 2016 Jul 6;108(11):djw153. doi: 10.1093/jnci/djw153 (PMC5241899; doi:10.1093/jnci/djw153)
Supplement: Supplementary Data [file supp_108_11_djw153__index.html]

Supplementary Data 

# Selenium and Prostate Cancer: Analysis of Individual Participant Data From Fifteen Prospective Studies

## Supplementary Data

files

- Supplementary Data - docx file
