# Supplementary material for: Selenium and Prostate Cancer: Analysis of Individual Participant Data From Fifteen Prospective Studies
Source: J Natl Cancer Inst. 2016 Jul 6;108(11):djw153. doi: 10.1093/jnci/djw153 (PMC5241899; doi:10.1093/jnci/djw153)
Supplement: Supplementary Data [file supp_djw153_16-0164R1_Allen_supp_mat_051016.docx]

**Supplementary Table 1. Details of the studies included**

| **Study** | **Country** | **Cohort description/comments** | | **Year of blood collection** | | **Prostate cancer ascertainment method** | **Cases:**  **Controls** | **Matching criteria** |
| --- | --- | --- | --- | --- | --- | --- | --- | --- |
| Blood selenium | | | | | | | | |
| BLSA (8) | USA | Population-based cohort study of predominantly men of white European ancestry (98%). Case-control sets were generated for this analysis. | | 1986-1997 | | Histological confirmation | 1:1 | Age (as close as possible), date of blood collection (as close as possible) |
| CARET (13) | USA | Randomised, placebo-controlled trial of retinol and β-carotene among heavy smokers, former heavy smokers and asbestos-exposed workers of predominantly men of white European ancestry (91%). | | 1985-1995 | | Histological confirmation of self-report (97%), unconfirmed self-report (2%), medical record linkage (1%), death certificate (<1%). Outcomes were adjudicated by three physicians using surgical, pathological and clinical records. | 1:2 | Age (5 yr categories), randomization year, smoking status, treatment arm, exposure population (asbestos/cigarette), year of blood draw |
| EPIC (17) | Europe | Population-based cohort study of men of white European ancestry. | | 1989-2000 | | Cancer registry linkage (Denmark, Italy, Netherlands, Spain, Sweden, UK), self-report with medical record review (Germany, Greece), | 1:1 (1:2 in Umeå cohort) | Study center, age at recruitment (±6 mths), time of day of blood collection (±1 hr), time between blood collection and last consumption of food or drinks (<3, 3-6, >6 hrs) |
| EPIC-Heidel (19) | Germany | Population-based cohort study of men of white European ancestry. Duplicate individuals who were also identified in EPIC were removed. | | 1994-1998 | | Self-report with medical record review | 1:2 | Age (5 yr categories), time of recruitment (6 mth categories) |
| FMC (15) | Finland | Population-based cohort study based on a screening examination of men of white European ancestry. | | 1968-1972 | | Cancer registry linkage | 1:2 | Municipality, age, point in time corresponding to cancer diagnosis |
| MEC (18) | USA | Population-based cohort study in men of different ethnic groups (13% of white European ancestry) | | 1994-2004 | | Cancer registry linkage | 1:2 | Geographic site, ethnicity, age at blood collection (±1 yr), date (±1 mnth), time of day of sample collection (±2 hrs), fasting status (<6, 6-7, 8-9, 10+ hrs) |
| NPC (2) | USA | Randomised trial of selenium supplementation on cancer in men of white European ancestry. Data provided for placebo arm only (case-control sets were generated for this analysis). | | 1983-1990 | | Medical record review | 1:3 | age at blood sampling (±1 yr), date of blood sampling (± 2 yrs) |
| PCPT (unpublished) | USA | Randomised trial of finasteride and prostate cancer (27) in predominantly men of white European ancestry (89%). Data provided for placebo arm only (case-control sets were generated for this analysis). All participants had a normal DRE and a PSA ≤ 3ng/mL. Selenium was measured in 2 pooled samples (taken at recruitment and year 4). | | 1993-1998 | | Prostate biopsy (all men) | 1:1 | Age at recruitment (as close as possible), date of recruitment (as close as possible), family history (where known). |
| PHS (10) | USA | Randomised trial of aspirin and β-carotene supplementation on heart disease and cancer in predominantly men of white European ancestry (94%). | | 1982-1983 | | Self-report with medical record review | 1:1 | Age (within 1 yr for men aged ≤55, within 5 yrs for men aged>55), smoking status |
| PLCO (16) | USA | Randomised trial of methods for early detection of cancer of the prostate, lung, colorectum and ovary in predominantly men of white European ancestry (84%). Data was provided for the screening arm only. | | 1993-2000 | | Self-report and medical record review | 1:1.2 | Age (5 yr categories), time since initial screening (1 yr interval), ethnicity, year of blood draw |
| SU.VI.MAX (unpublished) | France | Randomized trial of vitamin and mineral supplementation on risk of cancer and ischaemic heart disease (26). Case-control sets were generated for this analysis from the placebo arm only. Ethnic origin not stated. | | 1994-1995 | | Self-report and medical record review | 1:3-4 | Age (as close as possible), date of blood collection (as close as possible) |
| Nail selenium | | | | | | | | |
| CLUE II (14) | USA | | Population-based cohort study in predominantly men of white European ancestry (96%). | | 1989 | Cancer registry linkage | 1:2 | Age, ethnicity, date of participation (within 3 wks), size of toenail clipping |
| HPFS (11) | USA | | Cohort study of male health professionals in predominantly men of white European ancestry (94%). | | 1987-1988 | Self-report and medical record review | 1:1 | Age (±1 yr), smoking status, date of toenail return (within 1 mnth) |
| NLCS (12, 49) | The Netherlands | | Population-based cohort study in men of white European ancestry. Data was pooled from 2 analyses (12, 49), the most recent of which only included advanced cases and controls, resulting in a dataset of 2982 unique men. Case-control sets were generated for this analysis. | | 1986 | Medical record linkage | 1:1 | Age (as close as possible), length of follow-up (as close as possible) |
| SELECT (4) | USA | | Randomised trial of selenium and/or vitamin E supplement and risk of prostate cancer in men of white European ancestry (81%). Data was provided for the placebo arm only. All participants had a normal DRE and a PSA ≤ 4 ng/mL at study entry and underwent annual DRE and PSA measures and offered an end of-study biopsy. | | 2001-2004 | Self-report and medical record review, with central pathology review, where possible | 1:3 for black men, 1:1.5 for men of non-black ethnicity | Age (5 yr categories), ethnicity |

BLSA: Baltimore Longitudinal Study of Aging; CARET: the beta-Carotene and Retinol Efficacy Trial; CLUE II: Campaign against Cancer and Stroke ("Give us a Clue to Cancer") Study; DRE: Digital rectal examination; EPIC: European Prospective Investigation into Cancer and Nutrition; EPIC-Heidel: EPIC-Heidelberg; FMC: Finnish Mobile Clinic Health Examination Survey; HPFS: Health Professionals Follow-up Study; MEC: Multi-Ethnic Cohort; NLCS: Netherlands Cohort Study; NPC: Nutritional Prevention of Cancer trial; PCPT: Prostate Cancer Prevention Trial; PLCO: Prostate, Lung, Colorectal and Ovarian Cancer Screening trial; PHS: Physicians’ Health Study; PSA: prostate-specific antigen; SELECT: Selenium and Vitamin E Cancer Prevention Trial; SU.VI.MAX: SUpplémentation en VItamines et Minéraux Anti-oXydants trial.

**Supplementary Table 2. Assay details**

| **Study** | **Plasma or serum** | **Method** | **Assay and Laboratory** | **Within-batch CV** | **Between-batch or overall CV** |
| --- | --- | --- | --- | --- | --- |
| Blood selenium |  |  |  |  |  |
| BLSA (8) | Plasma | Atomic absorption spectrophotometry | Perkin-Elmer Corp,. Norwalk, Connecticut, US. Laboratory not stated | 2.9%, 2.2% and 2.0% at 4.8, 12.3 and 21.4 ug/dL, respectively | 11.7%, 8.7% and 9.1% |
| CARET (13) | Serum | Atomic absorption spectrophotometry | Perkin-Elmer Corp,. Norwalk, Connecticut, US. Laboratory not stated |  | not stated |
| EPIC (17) | Plasma | Inductively coupled plasma mass spectrometer | Elan DRCPlus (Perkin-Elmer Sciex) at the MRC Human Nutrition Research Unit, Cambridge, UK |  | 4.8% |
| EPIC-Heidel (19) | Serum | Inductively coupled plasma mass spectrometer | Elan DRCPlus (Perkin-Elmer Sciex) at Southampton University Hospital, UK | 2.9% to 4.4%. | 3.0% to 6.2% |
| FMC (15) | Serum | Atomic absorption spectrophotometry | Department of Biochemistry, National Public Health Institute, Finland |  | 3.0% and 6.8% |
| MEC (18) | Serum | Neutron activation analysis | University of Missouri-Columbia Research Reactor, Missouri, USA | 2.3% |  |
| NPC (2) | Plasma | Atomic absorption spectrophotometry | Perkin-Elmer Corp,. Norwalk, Connecticut, US at laboratory of Dr. Combs, Jr. |  | <7% |
| PCPT (unpublished) |  | Atomic absorption spectrophotometry | Perkin-Elmer Corp,. Norwalk, Connecticut, US. PHS Biomarker Lab, Fred Hutchinson Cancer Research Center, Seattle, USA |  | 2.4-12.4 |
| PHS (10) | Plasma | Neutron activation analysis | University of Missouri-Columbia Research Reactor, Missouri, USA |  | 6.4% |
| PLCO (16) | Serum | Inductively coupled plasma mass spectrometer | Dartmouth Trace Element Analysis Core Facility, Hanover, USA. |  | 9.4%. |
| SU.VI.MAX (unpublished) | Serum | Atomic absorption spectrophotometry | Perkin-Elmer Corp,. Norwalk, Connecticut, US. Laboratory not stated |  | Not stated |
| Nail selenium |  |  |  |  |  |
| CLUE II (14) | Toenail | Neutron activation analysis | University of Missouri-Columbia Research Reactor, Missouri, USA |  | 5% |
| HPFS(11) | Toenail | Neutron activation analysis | University of Missouri-Columbia Research Reactor, Missouri, USA |  | 2% |
| NLCS (12, 49) | Toenail | Neutron activation analysis | Subcohort: Snelle Buizen Post facility, Interfaculty Reactor Institute, Delft University, the Netherlands. Cases: Carbonfibre Autonomous Facility for Irradiation and Analysis, same institute as above.  Correlation coefficient of 40 samples measured in both facilities = 0.95 |  | 6.6% |
| SELECT (4) | Toenail | Neutron activation analysis | University of Missouri-Columbia Research Reactor, Missouri, USA | 2.8% | 3.0% |

**Supplementary Table 3.** Numbers of men with prostate cancer by selected characteristics in each study

| **Study** | **Number of cases** | **Years from sample collection to diagnosis** | | **Age at**  **Diagnosis** | | | **Year of**  **Diagnosis** | | **Stage of disease**^*^ | | | **Aggressive disease**^*^ | | | **Grade†** | | |
| --- | --- | --- | --- | --- | --- | --- | --- | --- | --- | --- | --- | --- | --- | --- | --- | --- | --- |
|  |  | **<5** | **5+** | **<60** | **60-69** | **70+** | **<1995** | **1995+** | **loc** | **adv** | **n/k** | **no** | **yes** | **n/k** | **Low-interm** | **high** | **n/k** |
| Blood selenium |  |  |  |  |  |  |  |  |  |  |  |  |  |  |  |  |  |
| BLSA (8) | 55 | 39 | 16 | 2 | 15 | 38 | 41 | 14 | 0 | 0 | 55 | 0 | 7 | 48 | 43 | 4 | 8 |
| CARET (13) | 235 | 165 | 70 | 33 | 132 | 70 | 50 | 185 | 118 | 50 | 67 | 146 | 28 | 61 | 175 | 28 | 32 |
| EPIC (17) | 959 | 605 | 354 | 187 | 623 | 149 | 4 | 955 | 497 | 203 | 239 | 515 | 238 | 206 | 639 | 102 | 218 |
| EPIC-Heidel (19) | 148 | 34 | 114 | 24 | 106 | 18 | 0 | 148 | 113 | 34 | 1 | 137 | 10 | 1 | 132 | 14 | 2 |
| FMC (15) | 51 | 23 | 28 | 5 | 21 | 25 | 51 | 0 | 0 | 0 | 51 | 0 | 0 | 51 | 0 | 0 | 51 |
| MEC (18) | 461 | 432 | 29 | 33 | 154 | 274 | 0 | 461 | 0 | 0 | 461 | 0 | 26 | 435 | 439 | 1 | 21 |
| NPC (2) | 41 | 23 | 18 | 0 | 18 | 23 | 38 | 3 | 31 | 10 | 0 | 34 | 7 | 0 | 34 | 6 | 1 |
| PCPT (unpublished) | 960 | 204 | 756 | 15 | 483 | 462 | 1 | 959 | 920 | 15 | 25 | 927 | 8 | 25 | 893 | 46 | 21 |
| PHS (10) | 794 | 120 | 674 | 95 | 354 | 345 | 610 | 184 | 634 | 119 | 41 | 592 | 166 | 36 | 686 | 82 | 26 |
| PLCO (16) | 723 | 643 | 80 | 40 | 404 | 279 | 0 | 723 | 630 | 93 | 0 | 673 | 50 | 0 | 676 | 43 | 4 |
| SU.VI.MAX (unpublished) | 100 | 28 | 72 | 34 | 66 | 0 | 0 | 100 | 0 | 0 | 100 | 0 | 0 | 100 | 84 | 10 | 6 |
| Nail selenium |  |  |  |  |  |  |  |  |  |  |  |  |  |  |  |  |  |
| CLUE II (14) | 117 | 79 | 38 | 10 | 43 | 64 | 86 | 31 | 63 | 31 | 23 | 76 | 22 | 19 | 100 | 8 | 9 |
| HPFS (11) | 181 | 114 | 67 | 20 | 84 | 77 | 180 | 1 | 94 | 79 | 8 | 98 | 79 | 4 | 110 | 29 | 42 |
| NLCS (12, 49) | 1,268 | 369 | 899 | 12 | 482 | 774 | 674 | 594 | 270 | 916 | 82 | 596 | 602 | 70 | 1154 | 11 | 103 |
| SELECT (4) | 404 | 318 | 86 | 51 | 221 | 132 | 0 | 404 | 396 | 0 | 8 | 393 | 3 | 8 | 333 | 19 | 52 |

^*^ Localised disease was defined as TNM ≤T2 and N0 or NX and M0, or equivalent (i.e. a tumor which does not extend beyond the prostate capsule); advanced stage was defined as T3 or T4 and/or N1+ and/or M1, or equivalent (i.e. a tumour extending beyond the prostate capsule and/or lymph node involvement and/or distant metastases), or unknown. Overall, 5315 (82%) of cases had data on stage. Aggressive disease was defined as T4 and/or N1+ and/or M1+, or Stage IV disease and/or death from prostate cancer. Overall, 5432 (84%) of cases had data on disease aggressiveness.

† Histological grade was categorized as low-intermediate grade (Gleason sum <8, or cases coded as well, moderately or poorly differentiated) or high grade (Gleason sum 8+ or cases coded as undifferentiated), or unknown. Overall, 5900 (91%) of cases had information on grade.

**Supplementary Figure 1.** Geometric mean (95% CI) of (A) blood, and (B) nail selenium concentration by baseline characteristics in control men, adjusted for study and age at blood collection

**(A) Blood selenium concentration (nmol/l) (B) Nail selenium concentration (ppm)**


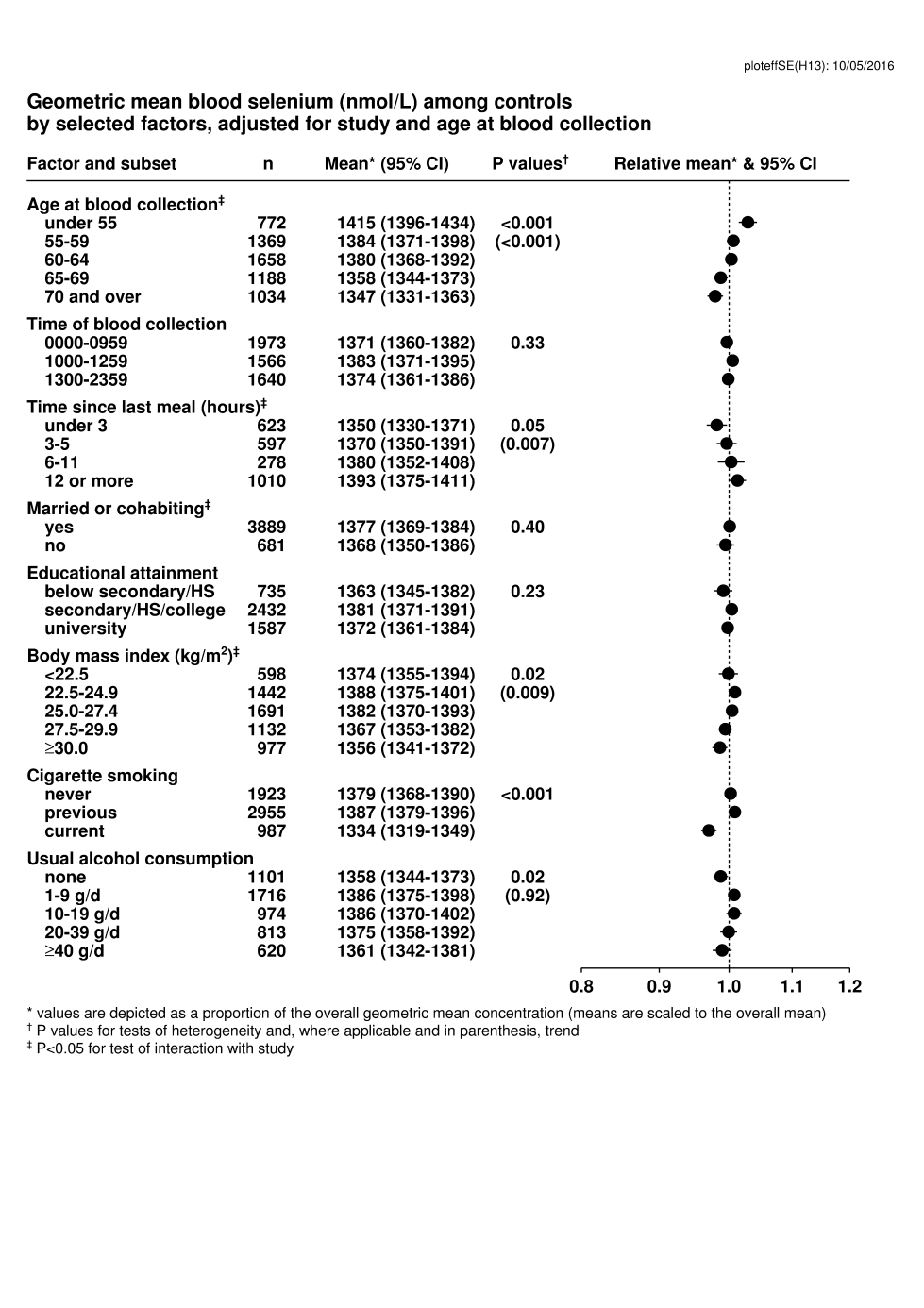

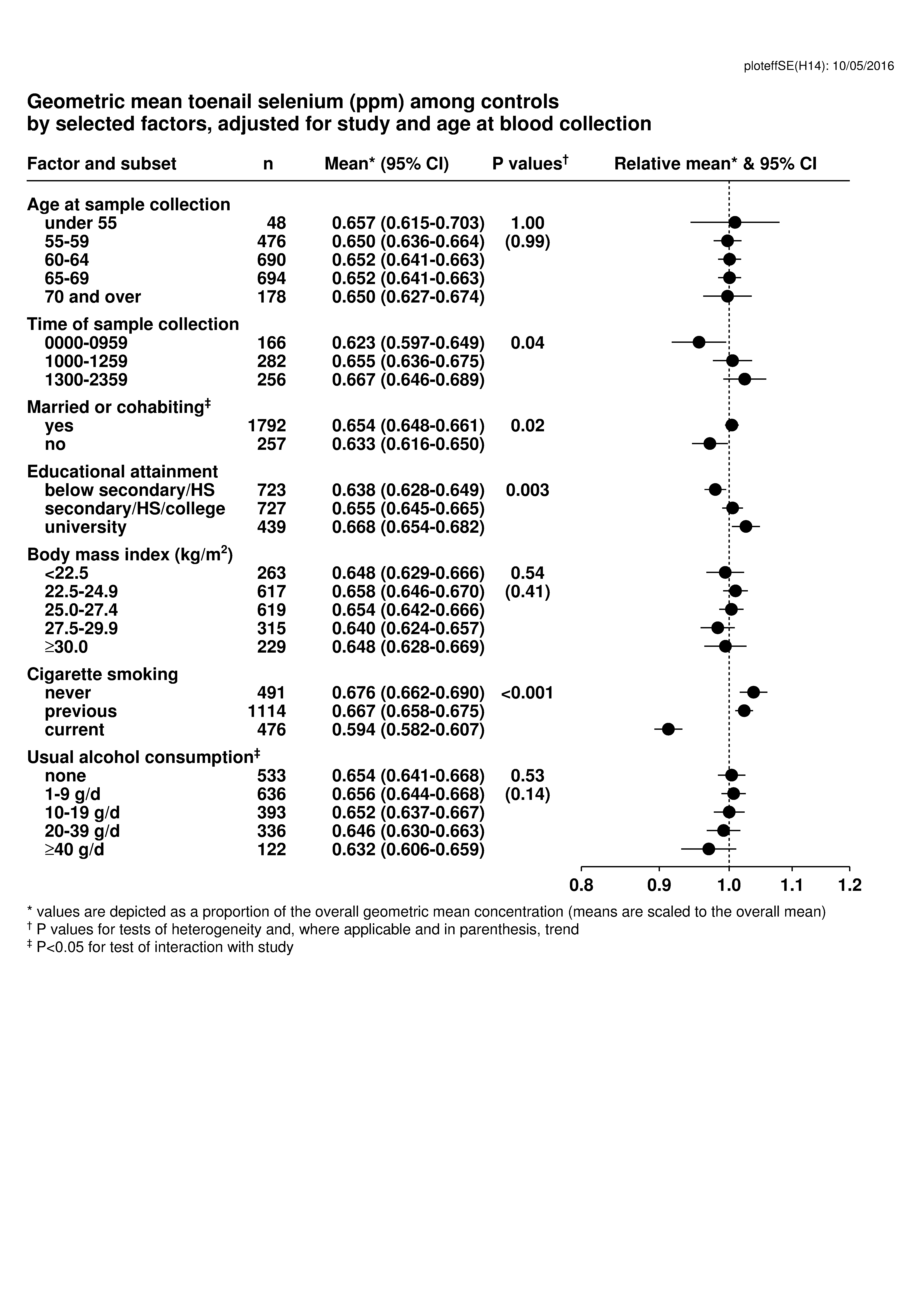


The figure for nail selenium (B) does not include time of blood collection or time since last meal as these factors are not applicable.

*Values are depicted as a proportion of the overall geometric mean concentration (means are scaled to the overall mean)

† P values for tests for heterogeneity and, where applicable and in parentheses, trend.

‡ P<0.05 for test of interaction with study.

**Supplementary Figure 2.** Odds ratio (95% CI) of prostate cancer associated with overall fifths of blood selenium by disease stage, aggressiveness and grade, adjusted for age, marital status, education, smoking, height and body mass index^a^

**
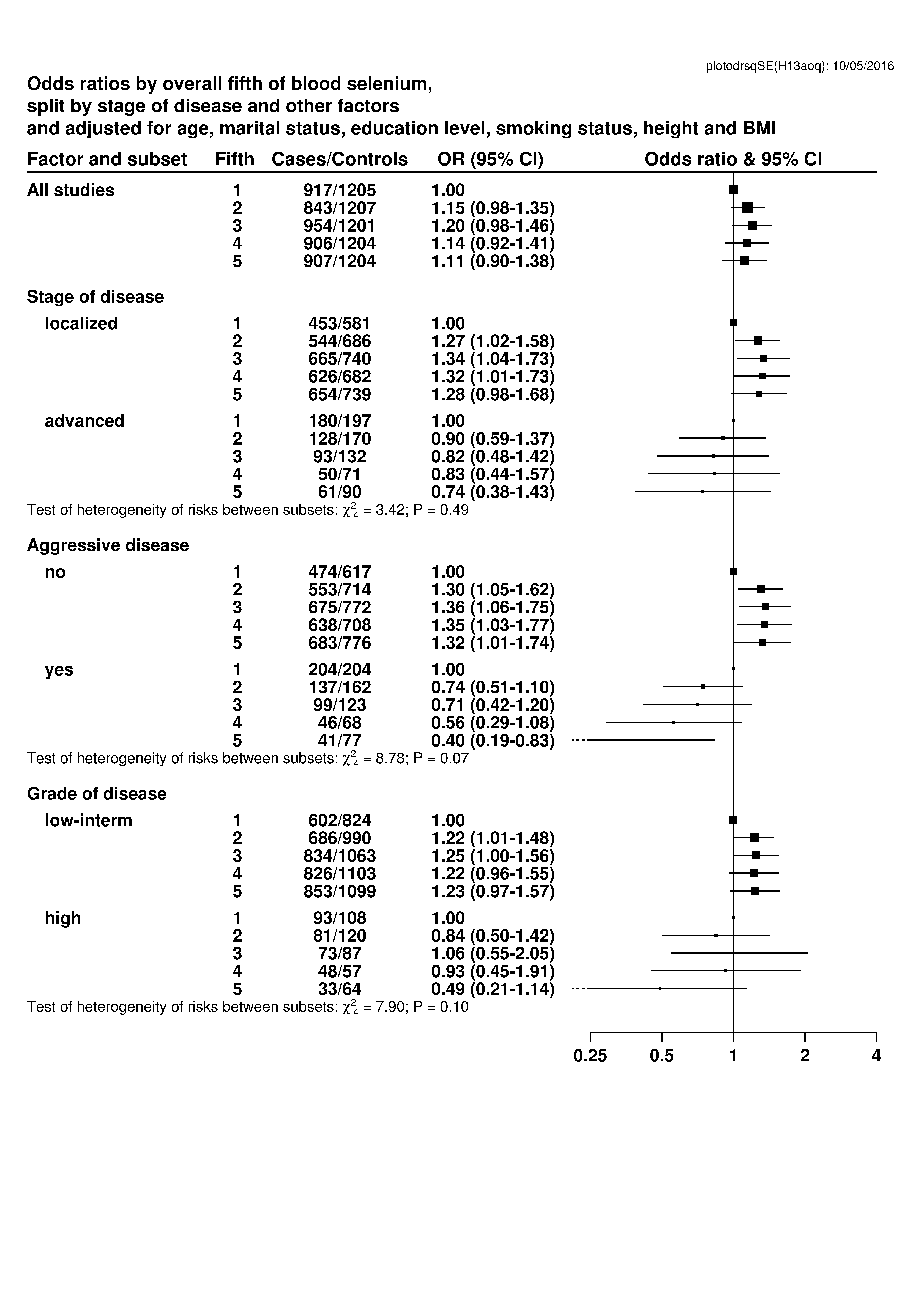
**

^a^ Tests for heterogeneity for the case-defined factors were obtained by fitting separate models for each subgroup and assuming independence of the ORs using a method analogous to a meta-analysis.
